# Unveiling the Potency of Gardenia Extract Against *H. pylori*: Insights from In Vitro and In Vivo Studies

**DOI:** 10.3390/biomedicines13010092

**Published:** 2025-01-02

**Authors:** Pornpen Werawatganone, Duangporn Werawatganon, Nattida Noonak, Maneerat Chayanupatkul, Tanittha Chatsuwan, Naruemon Klaikeaw, Walaisiri Muangsiri, Prasong Siriviriyakul

**Affiliations:** 1 Department of Pharmaceutics and Industrial Pharmacy, Faculty of Pharmaceutical Sciences, Chulalongkorn University, Bangkok 10330, Thailand; pornpen.w@chula.ac.th (P.W.); walaisiri.m@chula.ac.th (W.M.); 2 Center of Excellence in Alternative and Complementary Medicine for Gastrointestinal and Liver Diseases, Department of Physiology, Faculty of Medicine, Chulalongkorn University, Bangkok 10330, Thailandmaneeratc@gmail.com (M.C.);; 3 Department of Microbiology, Faculty of Medicine, Chulalongkorn University, Bangkok 10330, Thailand; 4 Department of Pathology, Faculty of Medicine, Chulalongkorn University, Bangkok 10330, Thailand; wnaruemon@gmail.com

**Keywords:** gastric inflammation, *Helicobacter pylori*, *Gardenia jasminoides* fruit extract, in vivo

## Abstract

Background and aim: *Gardenia jasminoides* (*G. jasminoides*) could treat various inflammatory diseases. This study aimed to investigate the effects of *G. jasminoides* fruit extract on gastric inflammation and protective mechanisms in *Helicobacter pylori* (*H. pylori*)-induced gastritis. Experimental procedure*: G. jasminoides* fruit extract was prepared and analyzed for geniposide content. The inhibitory effect of the extract on *H. pylori* growth was investigated using the disk diffusion method. The in vitro anti-inflammatory property of the extract was evaluated using the erythrocyte membrane stabilization method. Thirty-five male Sprague–Dawley rats were inoculated with *H. pylori* (10^8^–10^10^ colony-forming unit/mL) and divided into five groups. Each group was treated with various doses of the extract (98–395 mg/kg). The serum and stomach tissue of the rats were evaluated using enzyme-linked immunosorbent assay, histopathology, and immunohistochemistry. Results and conclusions: The geniposide content in the dried extract was 8.12% ± 0.79% by dry weight. The inhibition zone was observed at the extract ≥ 1.97 mg/disk, and the extract presented anti-inflammatory potential. The *H. pylori*-inoculated rats had a significant increase in serum interleukin (IL)-17, IL-33, and gastric epidermal growth factor (EGF) levels and a significant decrease in serum prostaglandin E_2_ level (*p* < 0.05) in conjunction with the development of gastric inflammation on histopathology. The treatment of the extract could significantly decrease the serum IL-17, IL-33, and gastric EGF levels, significantly increase the serum PGE_2_ level (*p* < 0.05), and improve gastric histopathology. Thus, *G. jasminoides* fruit extract attenuated *H. pylori*-induced gastritis by inhibiting bacterial growth, reducing inflammation, and enhancing protective mechanisms.

## 1. Background

*Helicobacter pylori* (*H. pylori*) is the main pathogenic bacterium involved in the development of gastritis, peptic ulcers, and gastric cancer [1]. *H. Pylori* is a gram-negative, spiral-shaped bacterium with a unique ability to colonize the gastric mucosa, especially at the antrum, and more than 50% of the world’s population have *H. pylori* infections [2]. The bacteria adapt to survive in the gastric mucosa by producing urease enzymes to neutralize the surrounding acidic environment and release virulence factors such as cytotoxin-associated gene A (CagA) and vacuolating cytotoxin A (VacA), which then translocate into the gastric epithelial cells, leading to the release of proinflammatory cytokines and enhancing levels of interleukin response to inflammation, thus causing gastric inflammation [3]. Furthermore, *H. pylori* colonization has a deleterious effect on mucosal integrity. Gastric mucosal damage could arise from the disruption of protective factors such as prostaglandins, mucus production, and bicarbonate secretion in the stomach [4].

*Gardenia jasminoides* Ellis (*G. jasminoides*) in the Rubiaceae family has been used as a traditional herbal medicine to treat various inflammatory diseases [5]. The extract of *G. jasminoides* fruit contains an iridoid glycoside called geniposide, which is a major bioactive component and is used as an indicator for the quantitative assay of *G. jasminoides* in Chinese pharmacopeia [6]. After the administration of geniposide, most geniposide is hydrolyzed to genipin by beta-glucosidase [7]. Geniposide and genipin exert several biological activities, including anti-inflammatory, antimicrobial, antioxidant, gastroprotective, and hepatoprotective properties [6]. Previous studies have demonstrated that geniposide and genipin reduced CagA, VacA, interleukin (IL)-8, interferon (IFN)-γ, and IL-1β expressions in *H. pylori*-infected mice [8]. Additionally, *G. jasminoides* extract decreased the levels of IL-6, IL-12, tumor necrosis factor (TNF)-α, and IFN-γ in HCl/ethanol-induced gastric injury in mice [9]. The aforementioned evidence suggested that *G. jasminoides* fruit extract might be useful in treating gastric inflammation.

In the present study, geniposide in the fruit extract was quantified, and the anti-inflammatory activities of the extract were studied in vitro and further investigated in vivo on *H. pylori*-infected rats. Accordingly, this study aimed to evaluate the protective mechanisms of *G. jasminoides* fruit extract after *H. pylori*-induced gastritis in rats.

## 2. Materials and Methods

### 2.1. Gardenia jasminoides Extract Preparation

Dried gardenia fruit was purchased from Vejpong Pharmacy Co., Ltd., Bangkok, Thailand. The plant material was identified by an experienced botanist, Dr. Chaisak Chansriniyom, and deposited at the herbarium of the Department of Pharmacognosy and Pharmaceutical botany, Faculty of Pharmaceutical Sciences, Chulalongkorn University, Thailand (voucher specimen number: CC-MS-0263). Ten grams of dried gardenia fruit was ground and soaked in 50 mL of water for 60 min. The filtrate of the fruit was collected. The crude was soaked again in 50 mL of 50% ethanol for 60 min and filtered. The filtrate from both steps was mixed and evaporated using an evaporator (R-300 BUCHI Rotavapor^®^, Flawil, Switzerland) [10]. The percent yield of the gardenia fruit extract was calculated using Equation (1). Geniposide in the dried extract was identified using liquid chromatography–mass spectrometry method. The detailed method was provided in Supplementary Materials. The content of geniposide in the evaporated extract was determined and the extract was kept at −20 °C until used for further study.

Percent yield = grams of dried extract × 100/grams of gardenia dried fruit(1)

### 2.2. High-Performance Liquid Chromatography (HPLC) Analysis

The geniposide content in the dried *G. jasminoides* extract was analyzed using the HPLC method modified from Chen et al. [11]. Geniposide standard was purchased from Sigma Aldrich, St. Louis, MO, USA. The separation of geniposide was performed on an HPLC system (Shimadzu LC20A^®^, Tokyo, Japan) equipped with a C18 Column (5 µm), 150 × 4 mm (Agilent^®^, Santa Clara, CA, USA), and an ultraviolet detector. The elution profile was 10% acetonitrile at 0 min, the linear gradient to 18% acetonitrile ranged from 0 to 15 min, the linear gradient to 28% acetonitrile ranged from 15 to 20 min, the linear gradient to 38% acetonitrile ranged from 20 to 40 min, the linear gradient to 50% acetonitrile ranged from 40 to 50 min, the final elution at 50% acetonitrile ranged from 50 to 55 min, and the linear gradient to 10% acetonitrile ranged from 55 to 64 min. The flow rate was 1 mL/min, and the injection volume was 10 µL. Geniposide was detected at 238 nm. (Supplementary Figures S1 and S2). This HPLC analysis method of geniposide was partially validated. Regarding Association of Analytical Communities (AOAC) 2016, specificity, accuracy, and precision values were evaluated and the results were in the acceptable range; accuracy (%recovery) of 98.44–97.99%, precision (%RSD) of 1.2760% (intraday) and 1.0452% (interday). The linear relationship of AUC and geniposide concentration was determined in the concentration range of 0.0050–0.1000 mg/mL at linearity of 0.9999.

### 2.3. In Vitro Tests

#### 2.3.1. Inhibitory Effect on *H. pylori* Growth

Antibacterial activities of *G. jasminoides* extract were examined using the disk diffusion method. Briefly, *H. pylori* suspension (10^8^–10^10^ CFU/mL) was spread onto the surface of Columbia agar. A sterile paper disk (6 mm in diameter) containing antibiotic amoxicillin (AML) 0.01 mg was used as a positive control. Furthermore, 10 μL of 0.5% methylcellulose (0.5% MC) was used as a negative control, and *G. jasminoides* extracts at 0.99, 1.97, 2.96, 3.94, 7.88, and 11.82 mg/disks were placed on the agar surface. Each sample was performed in triplicate. All agar plates were incubated under microaerophilic conditions at 37 °C for 72 h. The inhibition zone was measured in diameter (mm).

#### 2.3.2. Anti-Inflammatory Test

Anti-inflammatory activity was determined using the in vitro sheep red blood cell (SRBC) membrane stabilization method modified from previous studies [12,13]. Sheep blood was purchased from the Department of Animal Husbandry, Faculty of Veterinary Science, Chulalongkorn University, Thailand. The blood was centrifuged at 815× *g* (3000 rpm, MX-305; Tomy Seiko Co., LTD, Nerima City, Japan) for 20 min and washed three times with an equal volume of isosaline (0.85% *w*/*v* sodium chloride, NaCl). The volume of the sediment was measured, and it was reconstituted to 10% *v*/*v* SRBC suspension using isosaline.

The tested mixtures consisted of 2 mL of isosaline (0.85% *w*/*v* NaCl), 1 mL of 10 mM phosphate buffer saline mixed with *G. jasminoides* extract in the concentration range of 0.006–2.800 mg/mL, and 0.5 mL of 10% *v*/*v* SRBC suspension. The extract was replaced with diclofenac in the concentration range of 0.001–0.480 mg/mL and assigned as a positive control. The negative control was prepared in the same manner without the tested substances. All the tested mixtures were incubated at 56 °C for 45 min in which heat induced the lysis of the SRBC. Then, the tested mixtures were centrifuged at 3000 rpm for 20 min. The hemoglobin content in the supernatant solution was determined using a UV spectrophotometer (UV1601, Shimadzu, Japan), and absorbance was measured at 560 nm. Membrane stabilization or % hemolysis inhibition was calculated as follows (Equation (1)) at various concentrations of the tested substances:

(2) $$\%\;\mathrm{inhibition}\;\mathrm{of}\;\mathrm{hemolysis}=\frac{{\mathrm{absorbance}}_{\mathrm{negative}\;\mathrm{control}}-{\mathrm{absorbance}}_{\mathrm{sample}}}{{\mathrm{absorbance}}_{\mathrm{negative}\;\mathrm{control}}}\times 100$$

### 2.4. The In Vivo Study of Gastric Inflammation Induced by H. pylori

#### 2.4.1. Animals

Five-week-old male Sprague–Dawley rats were purchased from Nomura Siam International Co., Ltd., Bangkok, Thailand. The rats were allowed to acclimate to a new environment for at least one week in a room at 25 °C ± 1 °C under standard conditions with a normal 12-h light/dark cycle. All rats were fed a standard rat chow diet and sterilized tap water ad libitum. The experimental protocol was approved by the Ethical Committee of the Faculty of Medicine, Chulalongkorn University, Thailand (Approval No. 012/2562).

Rats were divided into five groups (*n* = 7 per group) as follows: control group (control), *H. pylori*-infected group (Hp), *H. pylori*-infected + extract 98.52 mg/kg (Hp + gen8) group, *H. pylori*-infected + extract 197.04 mg/kg (Hp + gen16) group, and *H. pylori*-infected + extract 394.09 mg/kg (Hp + gen32) group where 98.52, 197.04, and 394.09 mg of the extract contained 8, 16, and 32 mg of geniposide, respectively.

All rats had fasted for 16 h before the experiment. The *H. pylori*-infected rat model was previously described by Werawatganon [14]. Briefly, the rats were divided into four groups and were pretreated with streptomycin suspended in drinking water (5 mg/mL) for three consecutive days. Then, the rats were inoculated with 1 mL of *H. pylori* saline suspension (approximately 10^8^–10^10^ CFU/mL) twice daily at an interval of 4 h for three consecutive days using gastric gavage. Two weeks after *H. pylori* inoculation, three groups of the infected rats were fed with the extract suspended in 0.5% methylcellulose at the doses mentioned previously and once daily for one week by oral gavage. The control and *H. pylori* groups were fed with 0.5% methylcellulose once daily for one week. After the experiment, all rats were sacrificed with an intraperitoneal injection of sodium thiopental at a concentration of 50 mg/kg body weight.

#### 2.4.2. Gastric Histopathology

Hematoxylin and eosin staining was performed to determine the histopathology of gastric inflammation, and Giemsa staining was employed for *H. pylori* colonization. An experienced pathologist (N.K.) examined all blinded samples using a light microscope and determined the histopathological scores using the criteria of the updated Sydney System [15]. The gastric inflammation level was assessed and scored according to the degree of mononuclear and polymorphonuclear leukocyte infiltration in the gastric mucosa as follows: 0 = normal, 1 = mild inflammation, 2 = moderate inflammation, and 3 = marked inflammation. The level of *H. pylori* colonization was graded as follows: 0 = no bacteria detected, 1 = mild colonization, 2 = moderate colonization, and 3 = marked colonization.

#### 2.4.3. Enzyme-Linked Immunosorbent Assay (ELISA)

Serum samples were used to determine the levels of IL-17 (Novus Biologicals, Bio-Techne, CO, USA), IL-*33* (R&D Systems, Inc., Minneapolis, MN, USA), and PGE_2_ (R&D Systems, Inc., Minneapolis, MN, USA) using ELISA kits. Procedures were performed according to manufacturers’ protocols.

#### 2.4.4. Immunohistochemistry

Stomach sections were deparaffinized and the antigen was retrieved. Endogenous peroxidase activity and nonspecific binding were blocked with 3% hydrogen peroxide in methanol (Merck, Darmstadt, Germany) and goat serum (Dako, Carpinteria, CA, USA) diluted in PBS, respectively. Subsequently, sections were incubated with primary antibody for epidermal growth factor (EGF) at a dilution of 1:1000 (rabbit Anti-EGF antibody supplied by Novus Biologicals). The sections were then incubated with the secondary antibody (rabbit IgG horseradish peroxidase-conjugated antibody, R&D Systems, Inc.). The reaction was visualized by incubating the slides with diaminobenzidine. Finally, the sections were counterstained with hematoxylin.

Slides were scanned using the Aperio ScanScope System (Leica Biosystems Imaging, Inc., Silver Spring, MD, USA). The Aperio ImageScope software (v12.3.2.8013) was used to analyze and quantify marker expression. The gastric EGF expression was defined as gastric epithelial cells with brown-stained cytoplasm and cell membrane. The Aperio ImageScope Positive Pixel Count v9 algorithm was used for counting. Data were expressed as EGF positivity, which was quantified by the number of positive pixels over the total number of pixels by using a described method previously by Kalra et al. [16].

### 2.5. Statistical and Data Analysis

All data were presented as mean ± standard error of the mean. Continuous variables in each group were compared using a one-way analysis of variance with a least significant difference post-hoc test at *p*-value < 0.05. All statistical analyses were performed using the SPSS statistical program (version 22).

## 3. Results

### 3.1. Gardenia Jasminoides Extract Preparation

The obtained gardenia fruit extract was a yellowish flake, and the percent yield of the evaporated extract was 41.56 ± 0.96 by dry weight. Regarding HPLC analysis, the geniposide content of the extract was 8.12% ± 0.79% by dry weight. Previous studies have reported the percent yield of the extract at 41.4% and 4.03% of geniposide in the extract [17]. The percent geniposide in the extract was higher in the present study than in the previous study because of the difference in harvesting time, storage condition, and extraction process including method, solvent, temperature, and duration.

### 3.2. In Vitro Activity Tests

#### 3.2.1. The Inhibitory Effect on *H. pylori* Growth

The antibacterial activities of the extract were examined using the disk diffusion method. The extract inhibited *H. pylori* growth in a dose-dependent manner (Table 1 and Supplementary Figure S3). *H. pylori* growth was inhibited at the dose of 1.97 mg/disk and higher. Previous studies have demonstrated that *G. jasminoides* extract completely inhibited *H. pylori* colonization at a dose of 0.1 mg/mL [18]. The minimal bactericidal concentration (MBC) of geniposide against different *H. pylori* strains were 10 mM (3.88 mg/mL) [8]. Based on the calculation, the geniposide content was 0.16 mg in 1.97 mg of the extract. The difference in concentration could be due to different parameters that were measured. In this study, we measured minimal inhibitory concentration (MIC), not the MBC, which would require a higher dose of geniposide. Also, we used the total amount, not the concentration in the disk diffusion method. Nevertheless, following other studies, our findings showed that the extract was effective against *H. pylori*.

#### 3.2.2. Anti-Inflammatory Test

**Figure 1:**
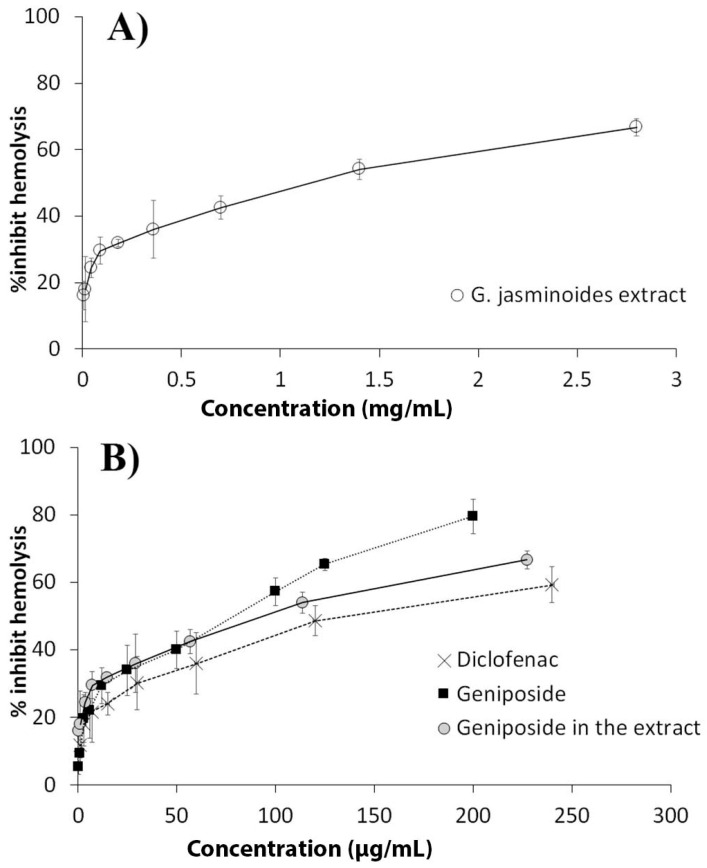
Correlation of hemolysis inhibition and concentration of tested substances using heat-induced hemolysis (n = 3); (**A**) *G. jasminoides* extract, (**B**) diclofenac, standard geniposide, and calculated geniposide in the extract.

Heat was used to induce hemolysis of SRBC. The SRBC ruptured in the negative control, leading to the presence of hemoglobin and UV absorbance at 560 nm. The ability of the extract to stabilize the cell membrane was presented as the percentage of hemolysis inhibition. The activities of the extract, geniposide, and diclofenac were plotted in Figure 1. The correlation lines were reported as percent hemolysis inhibition, the concentration of the tested materials in the extract was presented in Figure 1A, and geniposide and diclofenac were presented in Figure 1B. The increasing concentrations of these substances could enhance the stability of the cell membrane up to certain concentrations, and the activity subsequently plateaued. A high concentration range of the extract was needed to stabilize the cell membrane. The extract presented less anti-inflammatory potency because it contained some other inactive compounds. Based on geniposide content (8.12% ± 0.79% by dry weight), the geniposide concentration in the extract was calculated and plotted in Figure 1B. The standard geniposide had a higher potency than diclofenac. The potency of the extract corresponded to the geniposide content, and other ingredients in the extract likely caused deviation at high concentrations. The membrane of erythrocytes is considered an analog of lysosomes. The release of lysosomal constituents could induce tissue inflammation and damage. Stabilization of lysosomes likely limits the inflammatory response [12,19]. The extract could reduce cell lyses and potentially possess anti-inflammatory activity.

### 3.3. The In Vivo Study of H. pylori-Induced Gastric Inflammation

#### 3.3.1. Gastric Histopathology

**Figure 2:**
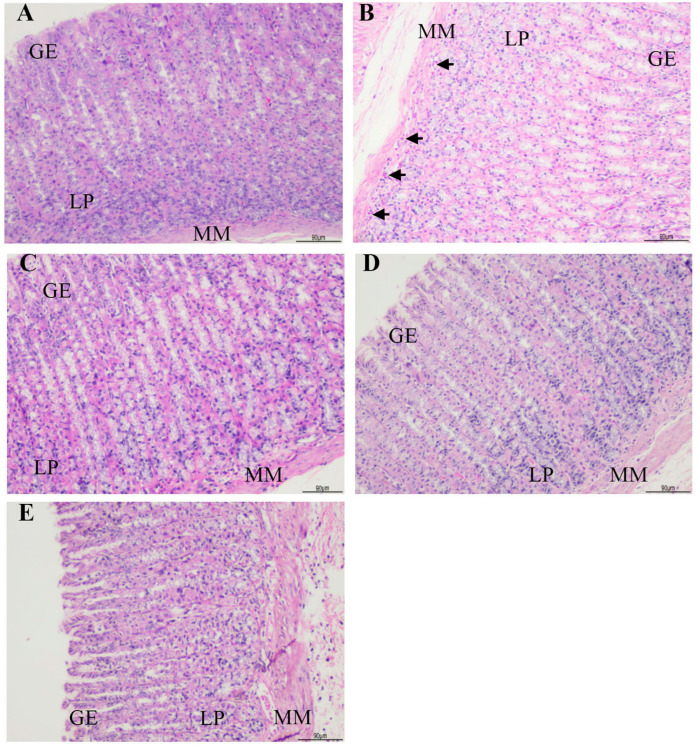
The histopathological examination of gastric inflammation in all groups; (**A**) Control group, (**B**) Hp group, (**C**) Hp + gen8 group, (**D**) Hp + gen16 group, (**E**) Hp + gen32 group, black arrows indicating inflammatory cell infiltration, GE = gastric epithelium, LP = lamina propria, MM = muscularis mucosae (H&E stain, 100× magnification, scale bar 90 µm).

**Figure 3:**
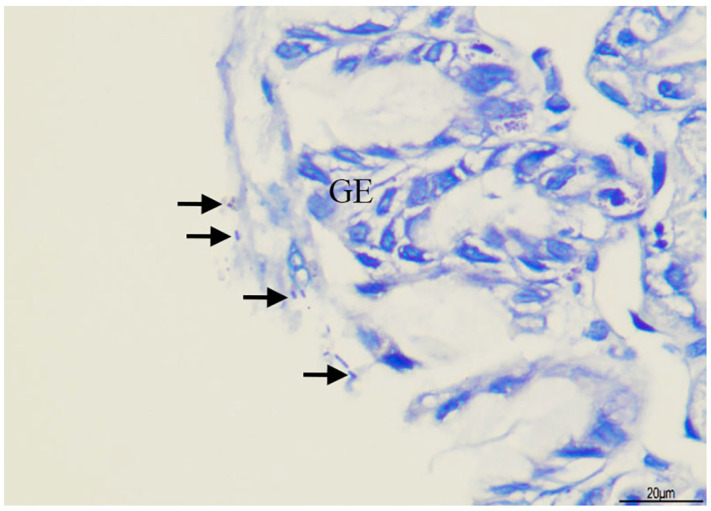
Histopathological examination of *H. pylori* colonization in Hp group; black arrows indicating *H. pylori* colonization. GE = gastric epithelium (Giemsa stain, 400× magnification, scale bar 20 µm).

The gastric histopathology of the rats is shown in Table 2 and Figure 2 and Figure 3. The control group presented normal gastric histopathology. Mild to moderate gastric inflammation along with *H. pylori* colonization was observed in the *H. pylori*-infected rats without treatment (Hp group). Less *H. pylori* colonization was found in the extract-treated groups, and improvement of gastric inflammation was also observed.

#### 3.3.2. ELISA

**Figure 4:**
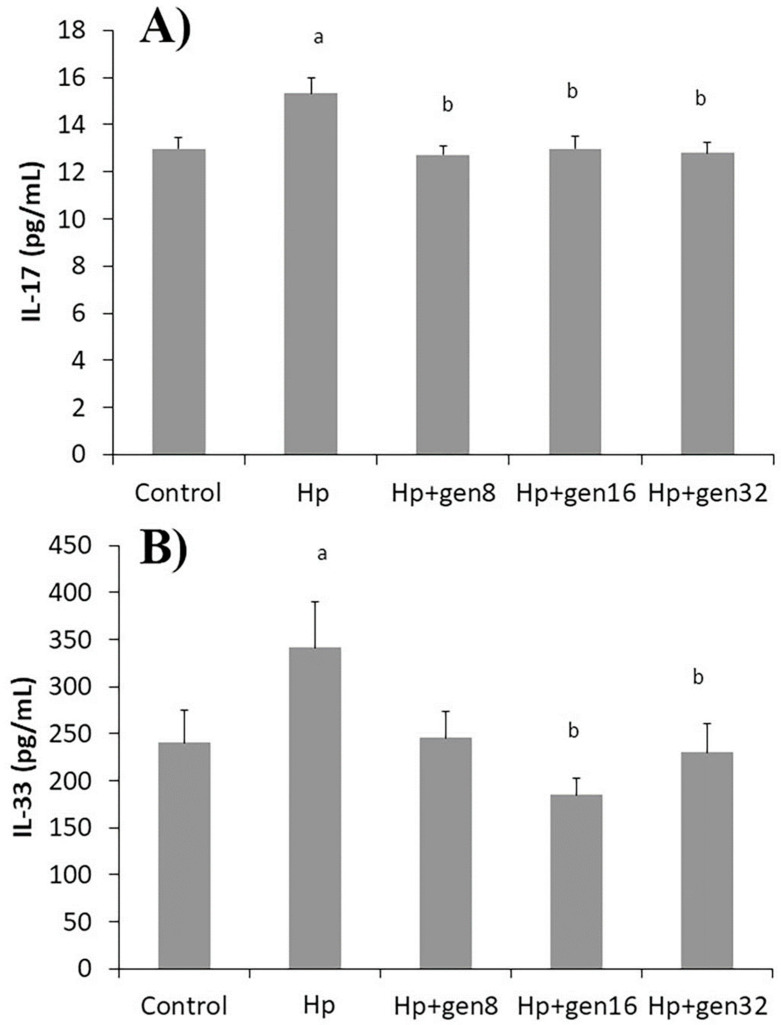
Effects of *H. pylori* and *G. jasminoides* extract on serum inflammatory cytokine levels in all groups; (**A**) serum IL-17 level, (**B**) serum IL-33 level (^a^ *p* < 0.05 vs. Control group, ^b^ *p* < 0.05 vs. Hp group).

Levels of both IL-17 and IL-33 in all rats were analyzed and reported in Figure 4. The serum IL-17 and IL-33 levels increased significantly in the Hp group when compared to the control group (*p* < 0.05). These cytokine levels of the control group were not significantly different from those of the extract-treated groups after *H. pylori* infection (*p* > 0.05). The cytokine levels of the extract-treated group at the dose of 197.04 mg/kg and higher were significantly less than those of the infected group without treatment (Hp group, *p* < 0.05). It is implied that this infection induced an inflammatory response in the gastric mucosa. This result corresponded to the gastric histopathology findings.

**Figure 5:**
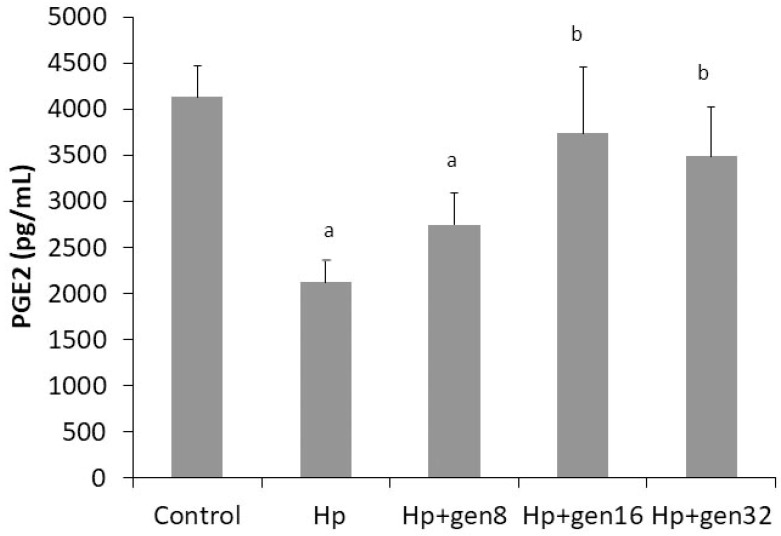
Effects of *H. pylori* and *G. jasminoides* extract on serum PGE_2_ in all groups (^a.^*p* < 0.05 vs. Control group; ^b^ *p* < 0.05 vs. Hp group.

Levels of serum PGE_2_ decreased significantly in the *H. pylori* and Hp + gen8 groups when compared with the control group (2124.29 ± 235.36 pg/mL vs. 2747.14 ± 345.61 pg/mL vs. 4130 ± 347.24 pg/mL, respectively, *p* < 0.05 for all comparisons). Serum PGE_2_ significantly increased in the Hp + gen16 and Hp + gen32 groups when compared with that in the *H. pylori* group (3740 ± 724.83 pg/mL vs. 3490.00 ± 532.81 pg/mL vs. 2124.29 ± 235.36 pg/mL, respectively, *p* < 0.05 for all comparisons) (Figure 5).

#### 3.3.3. Immunohistochemistry

**Figure 6:**
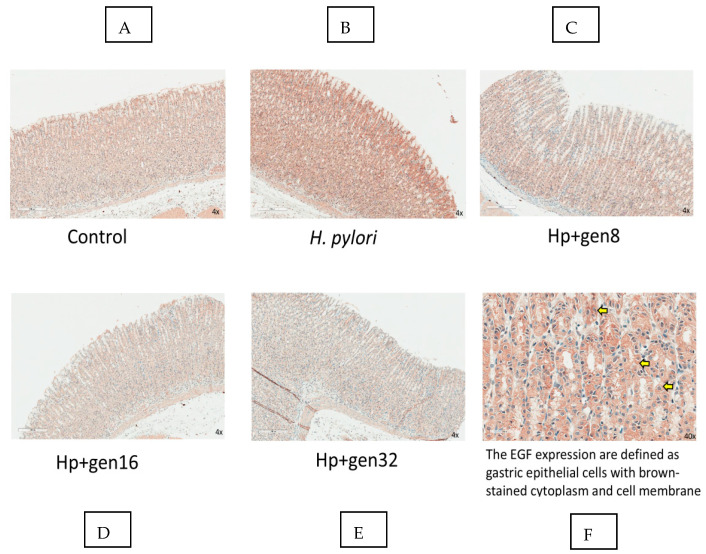
Immunohistochemistry of EGF expression; (**A**) Control group, (**B**) *H. pylori* group, (**C**) Hp + gen8 group, (**D**) Hp + gen16 group, (**E**) Hp + gen32 group, (40× magnification), and (**F**) *H. pylori* group with 400× magnification, yellow arrows indicating EGF expression.

**Figure 7:**
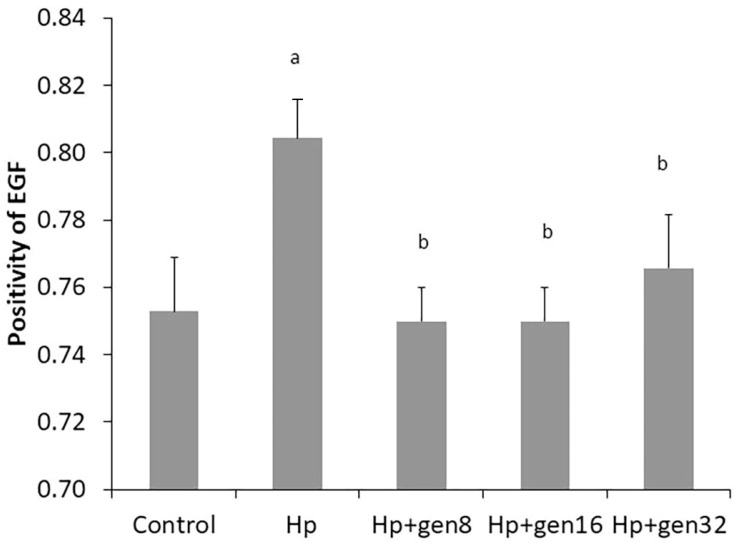
Effects of *H. pylori* and *G.jasminoides* extract on gastric EGF in all groups (^a^ *p* < 0.05 vs. Control group, ^b^ *p* < 0.05 vs. Hp group).

EGF expression and EGF positivity were presented in Figure 6 and Figure 7, respectively. *H. pylori* infection increased EGF expression as evidenced by the higher EGF positivity. EGF positivity increased significantly in the Hp group when compared with the control group (0.80 ± 0.01 vs. 0.75 ± 0.02, respectively, *p* < 0.05). The EGF positivity of the Hp group was significantly higher than that of the extract-treated groups after *H. pylori* infection.

## 4. Discussion

The mechanism by which *H. pylori* caused gastric inflammation was likely through its virulence factor, CagA. CagA stimulates dendritic cells to induce IL-17 expression from T helper 17 cells (Th17) [20,21,22]. IL-17 then stimulates gastric epithelial cells to produce inflammatory cytokines and chemokines such as IL-8, matrix metalloproteinase-9, and C–C motif chemokine ligand 25, leading to the recruitment of neutrophils and macrophages promoting gastric inflammation [23]. Several studies have reported that the levels of IL-17 increased in the gastric mucosa of both mice and patients infected with *H. pylori* along with the presence of mild to severe inflammation [20,24]. In the present study, the serum IL-17 levels increased in the Hp group, but the IL-17 levels in all the extract-treated groups after infection were not different from those in the control group. A previous study demonstrated that neutralization of IL-17 decreased bacterial colonization, inflammatory cytokines, and gastric inflammation [25]. Our results could be explained by the inhibitory effect of the extract on *H. pylori* growth, which reduced Th17 response and subsequently IL-17 production.

The virulence factors produced by *H. pylori* directly damage gastric epithelial cells together with the increase in inflammatory cytokine production. *H. pylori* releases virulence factor CagA that stimulates gastric epithelial cells to release IL-33 and induces inflammatory responses [26]. IL-33 is expressed in the nucleus of gastric epithelial cells, acts as a stomach alarmin, and is released to the extracellular environment after cell damage, stress, or necrosis [27]. Previous studies have presented that the serum IL-33 level increased in the gastric mucosa of mice and patients infected with *H. pylori* [27,28,29]. In the present study, the serum IL-33 level of the Hp group increased. The IL-33 increment was likely due to *H. pylori*-induced gastric epithelial cell damage. During *H. pylori* infection, gastric epithelial cell-derived IL-33 promotes TNF-α production leading to gastric inflammation and bacterial colonization. Conversely, the neutralization of IL-33 reduced TNF-α production, gastric inflammation, and *H. pylori* colonization [26]. Similarly, our results showed that treatment with the extract could reduce serum IL-33 in *H. pylori*-infected rats. Our results confirmed that the extract inhibited *H. pylori* colonization, thereby reducing proinflammatory cytokine production, inflammatory response, and cell damage.

The serum PGE_2_ level involved in the inflammatory response is reported in Figure 5. Cytokine expressions (IL-17 and IL-33) increased, whereas the serum PGE_2_ level decreased during *H. pylori* infection. The highest serum PGE_2_ level was observed in the control group, where the PGE_2_ level significantly decreased in the Hp and Hp + gen8 groups. The PGE_2_ level of the control group was not significantly different from those of the *H. pylori*-infected groups treated with the extract at a dose of 197.04 mg/kg and higher (*p* > 0.05). 

Prostaglandins are intrinsic mucosal defense mechanisms in the stomach. PGE_2_ is involved in the regulation of gastric mucosal inflammation and the modulation of mucosal integrity during *H. pylori* infection via the stimulation of mucus and bicarbonate production and secretion, increase in gastric mucosal blood flow, and inhibition of gastric secretion [30,31]. Therefore, PGE_2_ is considered a protective mechanism against gastric mucosal damage. Park et al. reported that in patients with both gastric and duodenal ulcers, the PGE_2_ levels were reduced in the active stage of ulcer disease and increased when the ulcer was healed [32]. In the Hp group, *H. pylori* infection reduced the serum PGE_2_ levels. Chen et al. demonstrated that patients with *H. pylori*-positive gastric low-grade intraepithelial neoplasia had lower levels of serum PGE_2_ than patients with *H. pylori*-negative LGIN [33]. Additionally, *H. pylori* increased the PGE_2_ synthesis in a gastric epithelial cell line that peaked at 24 h and declined at 48 h after *H. pylori* inoculation [34]. *H. pylori* infection increases the production of inflammatory mediators, disrupts intrinsic mucosal defense mechanisms, and impairs the cellular immune function of the gastric mucosa; some of these might be responsible for the reduction in the PGE_2_ level. The treatment of the extract could increase serum PGE_2_ after *H. pylori* infection. It is implied that the extract likely enhanced gastric mucosal protection against *H. pylori* by increasing the production of PGE_2_.

*H. pylori* infection is associated with increased gastric epithelial cell proliferation, thereby leading to gastric mucosal hyperproliferation that could promote the development of gastric cancer [4,35]. *H. pylori* colonization caused gastric mucosal damage resulting in the increased mucosal expression of EGF to promote the cell proliferative activity of the gastric mucosa and repair process. The increment of EGF expression occurred as a response to the increase in cell damage at the site of infection or damage caused by *H. pylori*. Previous studies have demonstrated that *H. pylori* infection increased EGF protein and epidermal growth factor receptor (EGFR) mRNA expression in patients with chronic active gastritis, gastric ulcers, duodenal ulcers, and gastric cancer [36,37]. This increased epithelial cell proliferation in *H. pylori*-induced chronic inflammation may play a role in its progression to gastric cancer.

In our study, the increment of EGF expression likely originated from gastric mucosa damage caused by *H. pylori* colonization. The treatment with the extract could reduce EGF expression in gastric mucosa. Clinical studies have shown that the levels of EGF and EGFR expression reduced after the eradication of *H. pylori* [36,38,39]. The reduction of EGF expression by the extract could be explained by the reduction of gastric inflammation and *H. pylori* colonization by this treatment.

## 5. Conclusions

This study showed that *H. pylori* infection induced gastritis by increasing gastric inflammation, IL-17, IL-33, and EGF production and decreasing PGE_2_ levels. In in vitro studies, *G. jasminoides* extract presented anti-*H. pylori* and anti-inflammatory activities. The anti-inflammatory effects of the extract were expressed well in the in vivo study by reducing IL-17, IL-33, and EGF levels, thus improving gastric histopathology. Moreover, *G. jasminoides* extract could enhance gastric protective mechanisms by increasing PGE_2_ levels. Further studies are warranted to determine the potential use of *G. jasminoides* extract as adjuvant therapy for *H. pylori*-induced gastritis in humans.

## Figures and Tables

**Table 1:** The inhibition zone diameters (mm) of different *G. jasminoides* extract dose.

Disk	Diameters of Inhibition Zone (mm) *
Amoxicillin 0.01 mg (positive control)	>59.64 ± 0.79
0.5%methylcellulose (negative control)	no inhibition zone
Extract 0.99 mg	no inhibition zone
Extract 1.97 mg	7.22 ± 0.11
Extract 2.96 mg	8.69 ± 0.04
Extract 3.94 mg	9.40 ± 0.10
Extract 7.88 mg	11.07 ± 0.03
Extract 11.82 mg	11.18 ± 0.02

* Diameters of blank disks = 6.00 mm.

**Table 2:** Summary of gastric histopathological scores in all groups.

Group	*n*	Gastric Inflammation *				*H. pylori* Colonization ^#^			
		0	1	2	3	0	1	2	3
Control	7	7	0	0	0	7	0	0	0
Hp	7	0	3	4	0	0	5	2	0
Hp + gen8	7	6	1	0	0	7	0	0	0
Hp + gen16	7	4	3	0	0	7	0	0	0
Hp + gen32	7	7	0	0	0	7	0	0	0

Figures indicate the number of rats with that score in each group. Histopathological scores using the criteria of updated Sydney System; (*) gastric inflammation score 0 = normal, score 1 = mild inflammation, score 2 = moderate inflammation and score 3 = marked inflammation, and (^#^) the level of *H. pylori* colonization score 0 = no bacteria detected, score 1 = mild colonization, score 2 = moderate colonization and score 3 = marked colonization.

## Data Availability

The data presented in this study are available on request from the corresponding author.

## Supplementary Materials

The following supporting information can be downloaded at: https://www.mdpi.com/article/10.3390/biomedicines13010092/s1 and Figure S1: HPLC chromatogram; Figure S2: Liquid Chromatography-Mass Spectrometer; Figure S3: The disk diffusion methods.

## List of Abbreviations

*H. pylori, Helicobacter pylori*; *G. Jasminoides, Gardenia jasminoides*; IL, interleukin; IFN, interferon; EGF, epidermal growth factor; EGFR, epidermal growth factor receptor; PGE2, prostaglandin E2; CFU, colony-forming unit; ELISA, enzyme-linked immunosorbent assay; MBC, minimal bactericidal concentration.
