# Supplementary material for: Unveiling the Potency of Gardenia Extract Against H. pylori: Insights from In Vitro and In Vivo Studies
Source: Biomedicines. 2025 Jan 2;13(1):92. doi: 10.3390/biomedicines13010092 (PMC11760463; doi:10.3390/biomedicines13010092)
Supplement: Supplementary file 1 [file biomedicines-13-00092-s001.zip › biomedicines-3348022-supplementary.pdf]

Supplementary Materials

The determination of geniposide content in the extract:
Geniposide in the extract was detected using liquid chromatography–mass spectrometry (LC-MS). The HPLC chromatogram revealed a geniposide peak at a retention time of 9.4 minutes

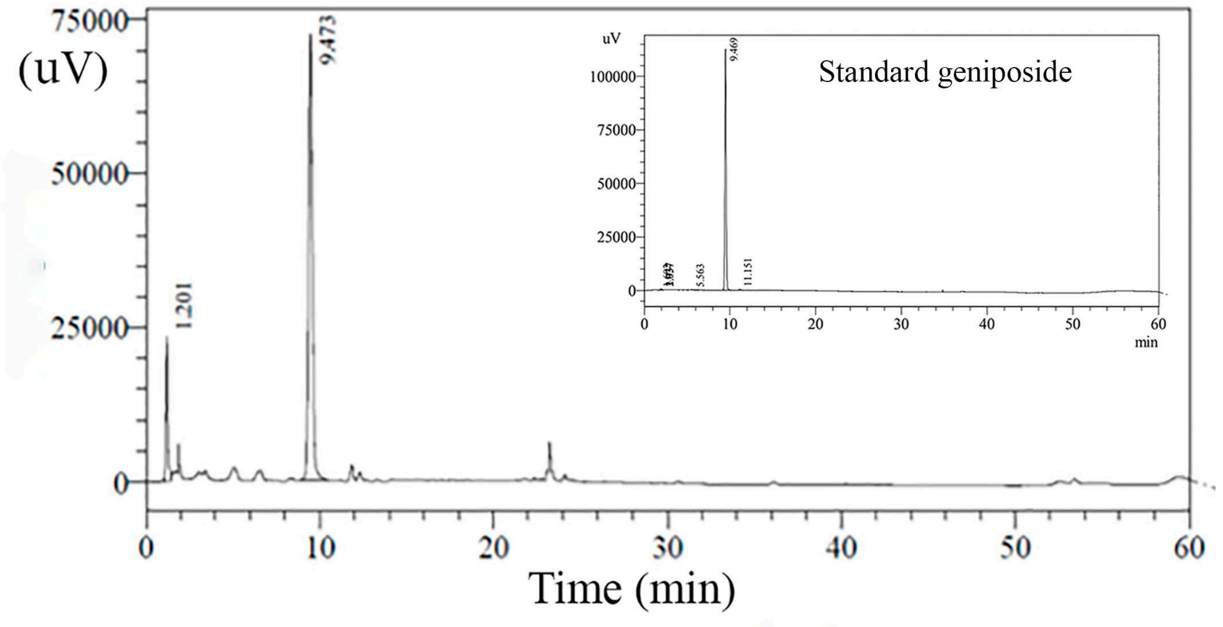

Supplementary Figure S1. HPLC chromatogram of G. jasminoides extract presenting geniposide detected at 238 nm and retention time of 9.47 min compared to the chromatogram of standard geniposide.

At this retention time, the mass spectra showed a molecular weight of 411.13, as displayed below.

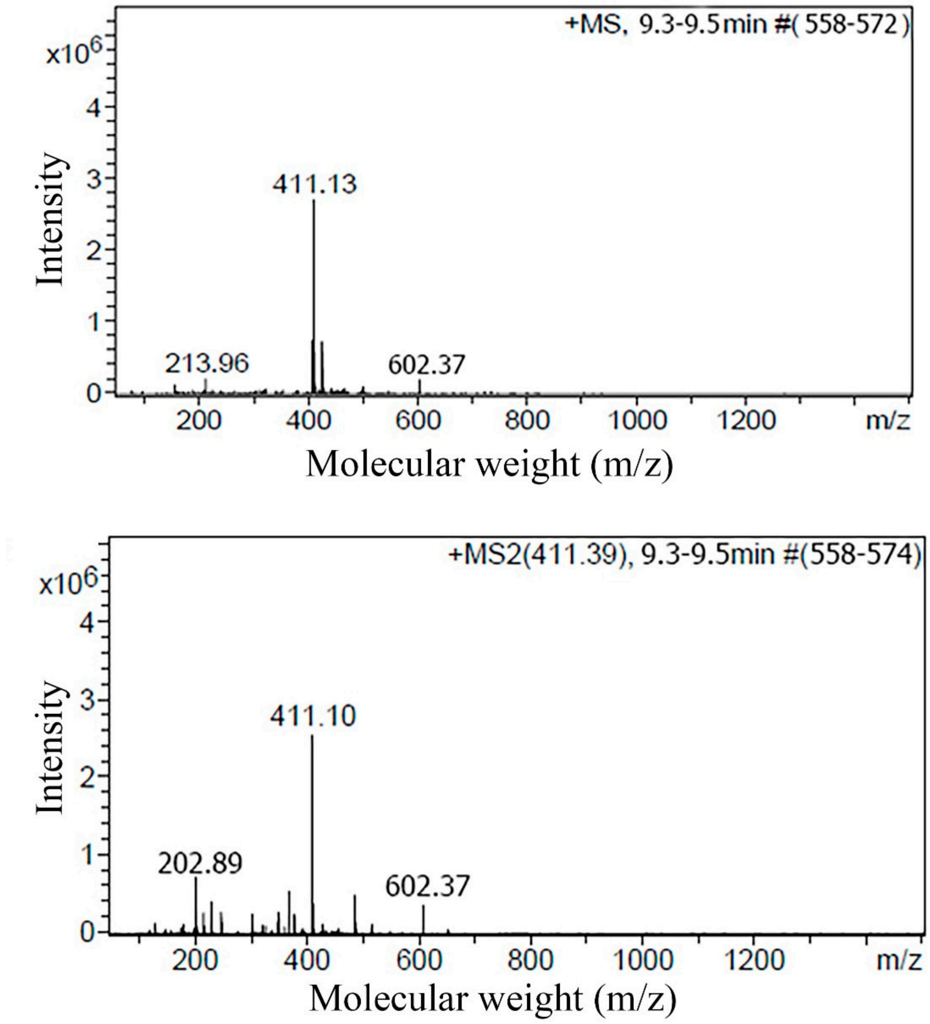

Supplementary Figure S2. Liquid chromatography–mass spectrometer of gardenia fruit extract mass spectra of the peak at a retention time of 9.4 min, molecular ions (MS) (a) fragment ions (MS^2) (b).

The results indicated that the 411 m/z peak in the gardenia extract corresponded to the combined molecular weight of geniposide and Na+. These findings align with previous studies, which reported MS2 spectra of geniposide at 203, 231, 249, and 379 m/z.

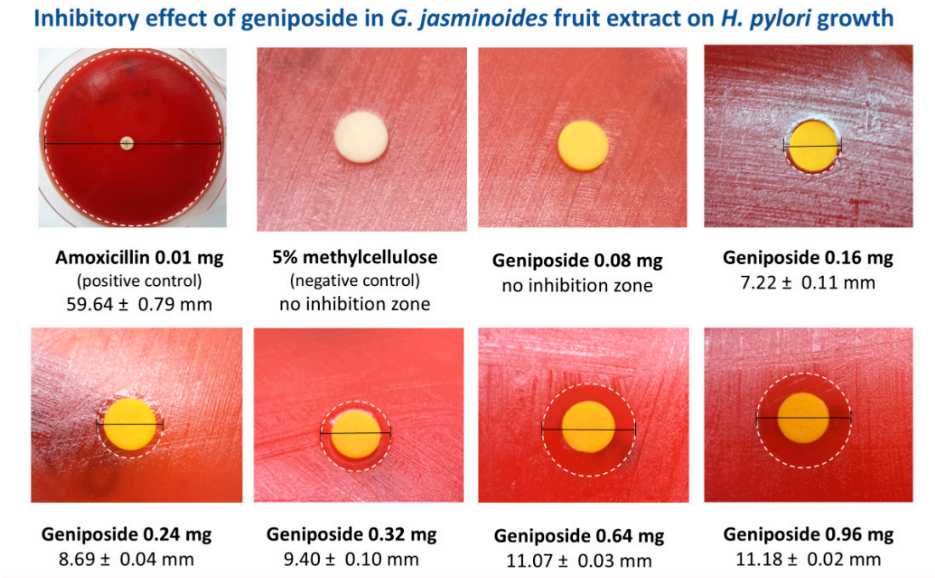

Supplementary Figure S3. Disk diffusion methods indicating the inhibition zone in the positive control, negative control, and various doses of geniposide (calculated from the extract).
